# Effects of the UV Filter Octocrylene and Its Degradation Product Benzophenone on Pacific Oyster (*Magallana gigas*) Larvae: A Call for Reassessment of Environmental Hazards

**DOI:** 10.3390/toxics13030177

**Published:** 2025-02-28

**Authors:** Ana Carvalhais, Romina Lippa, Isabel Benta Oliveira, Gaetano Di Lorenzo, Cláudia Mieiro, Mário Pacheco

**Affiliations:** 1 Centre for Environmental and Marine Studies (CESAM) and Department of Biology, University of Aveiro, 3810-193 Aveiro, Portugal; cmieiro@ua.pt; 2 Department of Biology, University of Perugia, Via Elce di Sotto 8, 06123 Perugia, Italy; lipparomina@gmail.com; 3 Interdisciplinary Centre of Marine and Environmental Research (CIIMAR), University of Porto, 4450-208 Matosinhos, Portugal; isabel_s_oliveira@yahoo.com; 4 Department of Biology, University of Naples Federico II, Via Cinthia 26, 80126 Naples, Italy; g.dilorenzo3099@gmail.com

**Keywords:** marine bivalves, embryotoxicity, swimming velocity, genotoxicity, cosmetics

## Abstract

Early life stages are pivotal to the functioning and resilience of ecological systems, displaying heightened vulnerability to environmental changes and exposure to contaminants. Octocrylene (OC), an organic ultraviolet (UV) filter, and its breakdown product benzophenone (BP) are commonly found in aquatic environments, but their impact on keystone processes determining the success or failure of the early life stages of marine organisms remains underexplored. This study aims to assess the impacts of OC and BP at environmentally realistic concentrations (1, 10, and 100 µg.L^−1^), over a 24 h exposure period, on larvae of the Pacific oyster (*Magallana gigas*). A multiparametric approach was employed, examining DNA integrity, embryo–larval development and swimming velocity. The results showed that DNA integrity and swimming velocity were not affected by OC or BP; however, both compounds increased developmental abnormalities in D-shaped larvae in all concentrations tested. Considering the robustness of morphological parameters, often assumed as irreversible, and their critical influence on larvae survival, these findings suggest that environmentally relevant concentrations of OC and BP may threaten the success of oyster larvae, potentially impacting the population’s long-term stability and, ultimately, raising ecological health issues.

## 1. Introduction

Reproduction and development are fundamental biological processes that support the functioning and stability of ecosystems. They are essential to ensure the continuation of species, maintenance of population sizes and genetic diversity. Therefore, understanding how environmental stressors affect those keystone steps is crucial to predict risks to populations and the potential impacts on the ecosystem health, as well as to drive conservation efforts. Furthermore, there is a consensus that the vulnerability to stressors can vary among the different developmental stages of marine species [1,2,3,4,5] and early life stages are considered more susceptible to environmental changes, such as human-induced disturbances [1,2,3,4,6]. Specifically, embryo–larval stages of marine bivalves demonstrated a high sensitivity to micro and nanoplastics [7,8], pesticides [9,10,11], metals [9,12], pharmaceutical [13] and personal care products [14,15]. Moreover, marine invertebrate larvae are easy to obtain and to grow in laboratory conditions, have a fast development, minimal ethical restrictions, and are use in standard embryotoxicity tests, making them highly suitable model organisms [16].

Ultra-violet (UV) filters are essential ingredients in cosmetics, one of the largest categories of personal care products, and are present in the formulations of sunscreens, creams, lipsticks, shampoos, and lotions. They are among the most commonly detected compounds on coastal surface water and have also been detected in sediments and biota [17,18,19,20,21]. Due to their high stability, lipophilicity and low biotic degradation, UV filters are bioactive and persistent in marine systems and have a high potential to bioaccumulate [18,22,23,24].

Octocrylene (OC) stands out as one of the most frequently used UV filters in cosmetics [17,25] and has been detected in seawater at concentrations between 0.026 and 172 µg.L^−1^ [26,27,28,29,30]. OC can induce harmful effects in aquatic organisms, mainly oxidative stress [31,32], endocrine disruption [33,34], and reproductive toxicity [35]. Moreover, its use as a UV filter was recently restricted as it was shown to have a potential risk to human health (EU No. 2022/1176) [36]. Recent studies showed that cosmetic products (e.g., sunscreens and day creams) containing OC also have benzophenone (BP) in non-negligible concentrations (even if pure BP itself is not included) [26,27,28,29,30], which is attributed to contamination during OC synthesis and to the degradation of OC into BP, a process that can occur both within the product over time [37,38,39] and potentially in the environment after the product is released. Medici et al. (2022) [39] reported that approximately 3% of OC degraded into BP within 2 h. Meanwhile, Downs et al. (2021) [37] investigated the degradation of OC in sunscreen product samples and observed a geometric mean increase in BP concentrations ranging from 38.7% to 199.4% after a 6-week accelerated stability incubation (equivalent to one year at room temperature).

As far as our knowledge goes, BP is not employed in cosmetics as a UV filter; rather, it serves as a UV light absorber, protecting products from UV light. Nevertheless, its use as an active ingredient in sunscreens is prohibited in some countries [40,41]. Somewhat surprisingly, as there are other sources of BP in the environment that are recognized (it is an ingredient of plastics, coatings, insecticides, agricultural chemicals, hypnotic drugs, antihistamines, and other pharmaceuticals) [41], there is a single study that reports a BP occurrence value in marine water [42], which, for the reasons we have mentioned, should not be considered representative. Anchored on the well-established OC degradation rates into BP, it can be hypothesized that this degradation product may occur in the aquatic environment at levels comparable to or even higher than the parental chemical.

According to the International Agency for Research on Cancer, BP is considered possibly carcinogenic to humans (Group 2B) (IARC, 2013) [41]. Information on its potential toxic effects on aquatic biota is notably scarce. In contrast, BP derivatives, commonly known as BP-type UV filters such as benzophenone-2 and benzophenone-3 (BP-3), are widely used as active sunscreen ingredients, with reported concentrations in marine environments ranging from 0.023 to 12.7 µg.L^−1^ [27,29,30]. These compounds are recognized for their detrimental effects on humans [43] and the environment [43,44,45] within their range of natural occurrence, including alteration of the amino acid metabolism in marine bacteria [46], gene alteration in anemones [47], DNA damage and bleaching in corals [48,49] and oxidative stress in turtles [50]. Due to these reported harmful effects, the European Union (EU) restricted their use in cosmetic products (EU No. 2022/1176) [36], and Hawaii (USA) banned cosmetics containing BP-3 to protect marine ecosystems [51].

Given the limited available information on the effects of OC (as a direct water contaminant and as a BP generator) on marine bivalves’ embryo–larval health, it is crucial to investigate the impacts of both parental and derivative compounds, contributing to an accurate assessment of their toxicity. In this context, the present study aimed to evaluate the potentially harmful effects of OC and BP on the early developmental stages of the Pacific oyster (*Magallana gigas*) within their (predictable, for the case of BP) range of environmental occurrence (1, 10 and 100 µg.L^−1^). This framework will provide detailed knowledge on potential subcellular effects, such as DNA integrity loss, and sub-individual/individual responses, including embryo–larval development and larvae swimming velocity. The Pacific oyster was chosen as a test species due to its sensitivity to environmental changes, wide distribution, easy sampling and analysis, and established protocols for its use [52].

## 2. Materials and Methods

### 2.1. Preparation of Octocrylene and Benzophenone Solutions

Octocrylene, 2-ethylhexyl 2-cyano-3,3-diphenylacrylate (declared purity ≥ 98% and CAS# 6197-30-4) and benzophenone (declared purity ≥ 99% and CAS# 119-61-9) were supplied by Merck (Rahway, NJ, USA). Both OC and BP stock solutions were freshly prepared in DMSO (dimethyl sulfoxide, declared purity = 99.8% and CAS# 67-68-5, supplied by Acros Organics (Antwerpen, Belgium)) with a concentration of 5 g.L^−1^. OC and BP exposure concentrations (OC1, BP1 = 1 µg.L^−1^; OC10, BP10 = 10 µg.L^−1^; OC100, BP100 = 100 µg.L^−1^ of OC and BP) were then prepared by diluting the stock solutions with artificial seawater (ASW, 30 salinity). The solvent control and all exposure media contained the same DMSO concentration of less than 0.002%, following OECD (Organization for Economic Co-operation and Development) guidelines, which recommend that the solvent concentration should not exceed 0.01% (OECD n121, 2010) [53].

### 2.2. Obtaining Oyster Gametes

Oysters were acquired in a local aquaculture (Ostraveiro, Aveiro, Portugal) during their reproductive season (May–July), and gametes were immediately collected based on ICES (International Council for the Exploration of the Sea) guidelines [54] by directly stripping the mature gonads of male and female oysters. Each gamete sample was filtered through a plastic mesh sieve (100 µm for oocytes and 64 µm for spermatozoa) and stored, until a fertilization procedure was carried out, in a clean glass test tube at room temperature.

### 2.3. Fertilization

Procedures were carried out according to the ICES oyster embryo–larval bioassay [54]. Briefly, within 30 min of obtaining the oocyte suspension (total volume: 100 mL; 6 × 10^3^ cells.mL^−1^; a pool of 3 females; agitation every 5 min), the sperm of 2 males was added in a ratio of 1:10 (oocytes:spermatozoa). Batches of abnormal oocytes, as well as immotile spermatozoa, were discarded. Fertilization success was verified under an optical microscope (Olympus BX50 (Tokyo, Japan)) until the embryos reached the 16–32 cell stage, and the obtained embryo suspensions were used for the toxicity tests described below.

### 2.4. Embryo–Larval Toxicity Tests

#### 2.4.1. DNA Damage

For genotoxicity evaluation, 20 mL of embryos (as obtained in Section 2.3) was placed into glass vials and exposed to OC or BP for 24 h at 24 ± 2 °C in the dark and without aerification to allow for embryo growth into D-shaped larvae. Dissociation of larval cells and comet assay were performed as described by Boukadida et al. [55], with some adaptations. Briefly, all the exposed larvae were recovered by sieving (25 µm) and resuspended in 1 mL of PBS. The concentration of larvae was adjusted to 12.000 per mL, and 500 µL of larvae suspension were incubated with 500 µL of Dispase II (1 g.L^−1^ in PBS) for 20 min at 37 °C, with gentle agitation every 5 min. The samples were centrifuged for 10 min at 1000 rpm and 4 °C to stop the reaction. The supernatant was removed, and the pellet resuspended with 400 µL of PBS.

Thereafter, 20 µL of cell suspension was mixed with 70 µL of 1% low-melting-point agarose. For each condition, 4 drops of 6 µL of cell suspension were placed onto the pre-coated slide. The slides were immersed into a lysis solution (2.5 M NaCl, 100 mM EDTA, 10 mM Tris and 1% Triton X-100, pH 10) at 4 °C for 1 h. Afterwards, the slides were placed horizontally in the electrophoresis tank with electrophoresis solution (0.3 M NaOH, 1 mM Na_2_–EDTA, pH > 13) for 20 min at 4 °C. Electrophoresis was conducted at 25 V, 300 mA for 20 min. The slides were rinsed three times in the neutralization buffer (0.4 M Tris, pH = 7.5) for 5 min at 4 °C, dehydrated in ethanol for 20 min and dried at room temperature. This assay was replicated 4 times.

For comet scoring, the slides were stained with SYBRGold (diluted 10,000×) for 30 min. Nucleoids were photographed using a Leica (Wetzlar, Germany) DM2000 LED fluorescence microscope (×100 magnification), and an average of 350 comets per treatment were scored with CometScore^TM^ software (v2.0.0.38, TriTek Corporation, Sumerduck, VA, USA). Genotoxicity was expressed through the percentage of tail DNA, represented by the amount of DNA breaks migrated from the nucleus.

#### 2.4.2. Embryogenesis Success

The assay consisted of embryo exposure, for 24 h, to 3 concentrations of OC and BP, *viz*. 1, 10 and 100 µg.L^−1^, along with a control (ASW, 30 salinity) and solvent control (ASW, 30 salinity and <0.002% DMSO) following the ICES oyster embryo–larval bioassay [54]. Normal development is defined as the transformation of embryos into straight hinge D-shaped larvae within the 24 h exposure period [6,54]. Embryos (as obtained in Section 2.3) were placed in a 24-well microplate in quadruplicates for each tested condition (100–200 embryos per well). Microplates with exposed embryos were incubated in the dark without aerification at 24 ± 2 °C. After incubation, the larvae were preserved with 37% buffered formalin (40 µL in each well). Unfertilized eggs were excluded from the analysis as they do not represent viable test organisms undergoing development [6,54]. The number of normal D-shaped and abnormal larvae was recorded for each test well under an inverted microscope (Leitz Labovert FS, Leitz, Wetzlar, Germany), and the percentage of abnormal D-shaped larvae was registered as a proxy for the inhibition of embryo development. Larvae were considered abnormal if they presented mantle or shell deformities and failed to reach the D-shaped stage (developmental arrest). This assay was replicated 3 times.

#### 2.4.3. Larvae Swimming Velocity

D-shaped larvae resulting from embryo exposure to OC and BP, described in Section 2.4.2, were used for larvae swimming velocity evaluation before preservation, according to Gamain et al. (2019) [56] and Bringer et al. (2020) [7]. Two 2 min videos were recorded [MOV format at 30 frames per second (fps)] in different places of each well (6 videos per condition) under an inverted microscope, with a magnification of 40×. VLC media player (v. 3.0.18) was used to subsample the videos to 6 fps and convert them into image sequences. All images were converted into grayscale using Matlab (R2023a, v.9.14.0.2306882) and then, into a binary stack of pictures using ImageJ (1.52a software). The entire stack of images was processed in the ImageJ plugin wrMTrck with the appropriate settings to track oyster D-shaped larvae [7,56]. Each tracked larvae (16–68 larvae per replicate) was assigned a different number, and the average speed (pixel/s) was calculated for swimming velocity analysis.

### 2.5. Statistical Analysis

A Student’s *t*-test was applied to compare the control with the solvent control in all endpoints studied. One-way ANOVA was used to assess the effect of independent variables (solvent control and the different treatments) on the dependent variables (larval abnormalities, swimming velocity and genotoxicity). Graphical validation tools verified ANOVA assumptions. A Dunnett’s post hoc test was performed to compare the different groups against the solvent control, ensuring that any observed differences were attributable to the test substances rather than the effects of the solvent itself. The results were presented as the mean and standard deviation. The statistical analysis was performed using IBM.SPSS^®^ (v27.0.1) and statistical significance was defined as *p* < 0.05.

## 3. Results

### 3.1. DNA Damage

**Figure 1:**
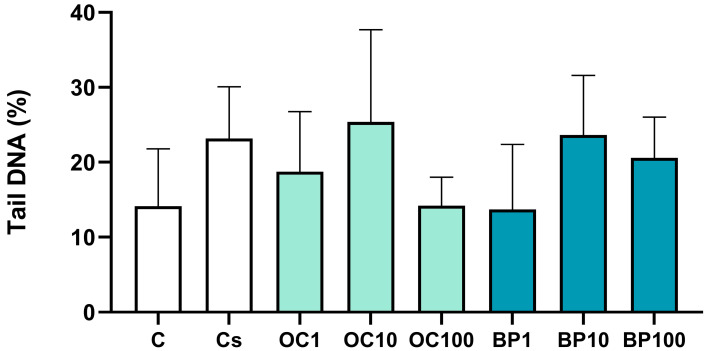
Genotoxicity measured by the comet assay as the percentage of the tail DNA in nucleoids of cells of the D-shaped larvae of the Pacific oyster (*Magallana gigas*) exposed to octocrylene (OC; OC1 = 1 µg.L^−1^; OC10 = 10 µg.L^−1^; OC100 = 100 µg.L^−1^) or benzophenone (BP; BP1 = 1 µg.L^−1^; BP10 = 10 µg.L^−1^; BP100 = 100 µg.L^−1^) (*n* = 4). Columns correspond to the mean values, and error bars represent the standard deviation. C—control; Cs—solvent control.

In this endpoint, the control and solvent control groups were statistically similar (t = −1.754, *p* = 0.130). No differences were observed among treatments and solvent control (F_(6,19)_ = 1.207, *p* = 0.345) (Figure 1).

### 3.2. Embryogenesis Success

**Figure 2:**
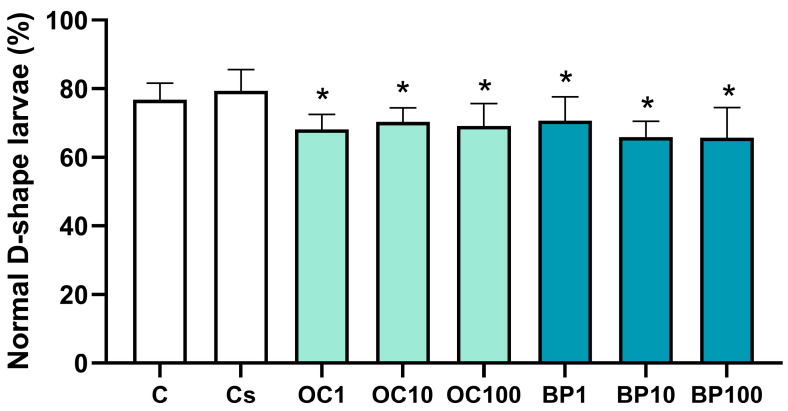
Embryo–larval toxicity assay translating the percentage of normal D-shape larvae of the Pacific oyster (*Magallana gigas*) exposed to octocrylene (OC; OC1 = 1 µg.L^−1^; OC10 = 10 µg.L^−1^; OC100 = 100 µg.L^−1^) or benzophenone (BP; BP1 = 1 µg.L^−1^; BP10 = 10 µg.L^−1^; BP100 = 100 µg.L^−1^) over 24 h (*n* = 12). The asterisks reflect significant differences between the treatments and solvent control (*p* < 0.05). Columns correspond to the mean values, and error bars represent the standard deviation. C—control; Cs—solvent control.

After 24 h of exposure, the percentage of normal D-shaped larvae decreased significantly (about 11%) in all treatments compared to the solvent control (F_(6,76)_ = 6.688, *p* < 0.001) (Figure 2). No significant differences were observed between the control and solvent control concerning the frequency of normal D-shaped larvae (t = −1.163, *p* = 0.257).

**Figure 3:**
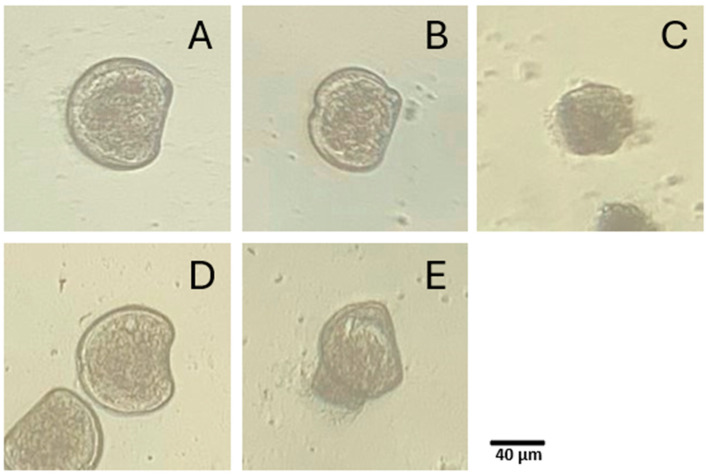
Photomicrographs of D-shaped larvae of the Pacific oyster (*Magallana gigas*) following 24 h of development: (**A**) normal shape, (**B**) indented shell margin, (**C**) developmental arrest, (**D**) convex hinge and (**E**) protruding mantle.

Four types of anomalies were observed in D-shaped larvae after exposure to OC and BP (Figure 3). In this direction, indented shell margin (shell deformity) (Figure 3B) was the predominant abnormality observed. Developmental arrest (Figure 3C), a convex hinge (shell deformity) (Figure 3D) and a protruding mantle (Figure 3E) were also occasionally observed.

### 3.3. Swimming Velocity

**Figure 4:**
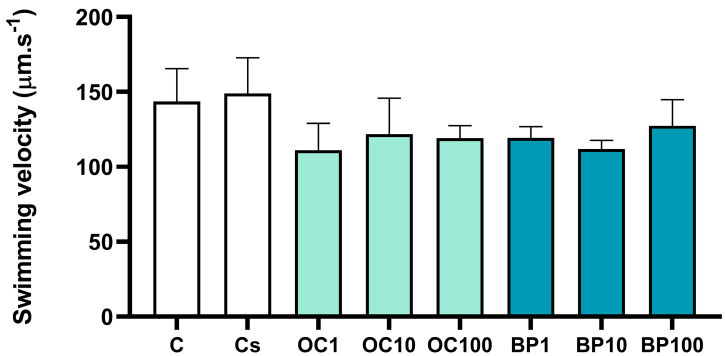
Swimming velocity of the D-shaped larvae of the Pacific oyster (*Magallana gigas*) after 24 h of exposure to octocrylene (OC; OC1 = 1 µg.L^−1^; OC10 = 10 µg.L^−1^; OC100 = 100 µg.L^−1^) or benzophenone (BP; BP1 = 1 µg.L^−1^; BP10 = 10 µg.L^−1^; BP100 = 100 µg.L^−1^) (*n* = 3). Columns correspond to the mean values, and error bars represent the standard deviation. C—control; Cs—solvent control.

This endpoint was similar in the control and solvent control groups (t = −0.283, *p* = 0.791) with a swimming velocity average of 143.5 ± 22.1 µm.s^−1^ and 148.9 ± 23.8 µm.s^−1^, respectively. After 24 h of exposure, no significant differences in swimming velocity were observed between the solvent control and the OC and BP treatments (F_(6,14)_ = 1.783, *p* = 0.175) (Figure 4).

## 4. Discussion

It is well accepted that early life stages of bivalves show greater sensitivity to environmental disturbances than the adult stages [1,2,3,4,5] due to biochemical, morphological, physiological, and behavioral characteristics. For example, larvae exhibit a larger surface area of permeable membrane compared to more advanced developmental stages, which is critical for the uptake of toxicants. Moreover, early life stages are not fully prepared to metabolize and eliminate xenobiotics as efficiently as adults and the time required for a toxicant to reach a structure is shorter in larval stages due to their smaller size and incompletely developed organs [4]. Furthermore, adults can improve their resilience, actively reducing the time of exposure to a toxicant by closing their valves for a certain period of time [57] as well as chelating a variety of toxic substances in specific proteins and fat tissue so that other target organs are protected [4]. Taking together the previously mentioned features and the direct influence of survival and health of early life stages on population dynamics, the impact and novelty of the present study in terms of mechanistic understanding of UV filters toxicity and its ecological relevance are highlighted. These merits are reinforced by the adoption of a multi-level diagnosis, combining the assessment of DNA damage, morphological alterations, and behavioral shifts, thereby integrating molecular damage with structural and functional consequences at the organismic level.

Genome integrity and stability are crucial for organisms’ health, fitness and, ultimately, survival [58]. The available literature points towards a genotoxic potential of BP-type UV filters [59,60], reporting DNA fragmentation in single-cell green algae (*Chlamydomonas reinhardtii*) after 24 h of exposure to BP-3 (2.5 × 10^3^, 5 × 10^3^ and 10 × 10^3^ µg.L^−1^) and BP-4 (19 × 10^3^, 38 × 10^3^ and 76 × 10^3^ µg.L^−1^) [60], as well as DNA damage in blood cells of the guppy fish (*Poecilia reticulata*) after exposure to BP-3 (0.1 and 1 µg.L^−1^) [59]. On the contrary, in our study, BP did not induce DNA damage. These discrepancies may be related to (i) the higher concentrations used in the referenced studies compared to those in our study (1 to 100 µg.L^−1^), (ii) the differences in the tested species and (iii) the type of cells exposed, which may exhibit different sensitivities to genotoxic agents. A similar results profile was observed for OC. This result is in line with the detection of an upregulation of the DNA damage repair marker (GADD45) in adult mussels (*Mytilus edulis*) exposed to OC at 10 and 100 µg.L^−1^ for 14 days [32].

Larval abnormalities are one of the most used criteria in embryo–larval ecotoxicological tests [6]. Regarding the ability of OC and BP to induce developmental abnormalities, the current study’s data revealed that the oyster D-shaped larvae’s morphology is negatively affected by the exposure of embryos to these compounds in all concentrations tested. To the best of our knowledge, there is no previously published information on the effects of OC or BP on bivalves’ larvae. However, similar results were reported in zebrafish embryos exposed to environmentally relevant concentrations of OC (50 and 500 µg.L^−1^), showing developmental abnormalities [31]. Moreover, chronic exposures to OC (5, 50 and 500 µg.L^−1^) also increased developmental malformations in Japanese medaka embryos [35]. No information for BP is available, yet, for other BP-type UV filters, namely, BP-3 and BP-8, a study showed almost no malformations in zebrafish embryos after exposure to 1, 30 and 300 µg.L^−1^ [61].

In the present study, the most frequent anomaly in D-shaped larvae occurring as a consequence of OC and BP exposure was a shell deformity (indented shell margins). Previous studies showed that the early shell formation of Pacific oysters is affected by human-induced stressors, such as pesticides, metals, and polycyclic aromatic hydrocarbons [9,12,62]. The biogenesis of the primary shell of bivalves, including *M. gigas*, is attributed to tyrosinase genes, which seem to be a target for various environmental stressors in larval stages until D-shaped larvae [16,63,64,65], and appears to be involved in counteracting the damage in shell formation during ocean acidification [63]. Hence, it can be hypothesized that OC and BP may affect tyrosinase gene expression, impairing shell formation. Additionally, shell formation depends on endogenous energy since larvae lack developed feeding organs. D-shaped oyster larvae may require 30% of energy from the oocyte reserves, mainly lipids, to properly form their shell, and protein synthesis for the shell organic matrix has a high energy cost [66]. Exposure to OC and BP may also interfere with the energy budget of larvae, making metabolic processes more energetically expensive. Therefore, realistic concentrations of OC and BP induce abnormalities in D-shaped larvae and such sub-lethal effects can affect their normal development and lead to an extension of larval stage. Moreover, the survival rates of larvae with shell deformities can be reduced as such abnormalities may prevent them from successfully undergoing metamorphosis in nature [67].

Larval behavior is a suitable endpoint for assessing the impact of human-induced stressors [56] since it reflects a cumulative integration of multiple physiological and neuromotor processes, offering insights into fitness and ecological performance. Previous studies reported OC and BP-type compounds as neurotoxic for zebrafish [68], but there is no information about bivalves. Neuroactive substances can modify swimming behavior in veliger larvae [69] and, at this stage, D-shaped larvae already have a serotonergic system, which is responsible for vital functions (e.g., swimming) [16] and can be a target for the toxic action of the tested UV filters. Abnormal swimming behavior might drastically affect larvae survival, leading to increasing larvae predation, infection, mortality and delayed settlement, which may be translated into a reduction in oyster recruitment [3,5,7,56]. Under the currently assayed conditions (24 °C, salinity of 30), the average velocity of oyster D-shaped larvae in control and solvent control was 143.5 and 148.9 µm.s^−1^, respectively. These velocities are similar to those reported by Gamain et al. (2019) [56] for unexposed *M. gigas* D-shaped larvae (144 µm.s^−1^ at 24 °C, salinity of 33). To our knowledge, there are no studies linking these or similar compounds with bivalve larval behavior. Like in our study, Gamain et al. [56] found that copper and S-metolachlor did not affect oysters’ swimming velocity, suggesting that these compounds do not target D-larvae’s metabolism or neuromuscular system. However, they observed disrupted swimming trajectories, proposing that circular swimming patterns observed in developing oyster embryos may signal the development of morphological anomalies. Due to methodological constraints, swimming trajectories were not registered, but based on the findings of Gamain et al. [56], we hypothesize that similar swimming pattern shifts may occur, given the morphological alterations in larvae at all tested concentrations, warranting further investigation.

OC, an unstable ingredient in cosmetics, degrades into BP, one of the most toxic of its byproducts [37,38,39,70]. Our results suggest that both OC and BP impair the early life stages of Pacific oysters at environmentally relevant concentrations, causing embryotoxicity. These findings confirm the sensitivity of oyster larvae to UV filter contamination but also highlight the need for further research to enable a more in-depth understanding of their toxicological mechanisms.

Given the sensitivity of early life stages, we recommend following standardized testing guidelines (e.g., ICES, 2013) to ensure adequate environmental protection. Additionally, investigating the parent compounds and their degradation products, which can occur in both the product and the environment, is crucial, as they may have distinct and potentially more significant toxicological impacts.

Endpoints at lower levels of biological organization, such as DNA integrity, are highly sensitive and provide early warning sublethal effects, enabling effective hazard prediction [71]. In contrast, higher-level responses, like morphological and behavioral assessments, are less sensitive at an early stage but more indicative of ecological and functional health [72]. While DNA-level effects may be reversible and not always translate to observable harm, individual-level impacts are typically permanent and can influence fitness and population dynamics. A holistic approach, encompassing lower and higher biological levels, is essential [71,72]. However, higher-level endpoints are often more suitable for setting practical exposure limits, as they reflect observable adverse effects on individual organisms and population outcomes [72], ensuring the findings are actionable and directly applicable to policy and management, as demonstrated by the data from the embryotoxicity success.

The contamination of estuarine systems with OC and BP may negatively impact oyster recruitment, with potential cascading effects on other bivalves, especially native species. Reduced recruitment could lead to decreased bivalve reefs, habitat loss, diminished coastal protection, and economic consequences for local communities. Overall, the depletion of bivalve recruitment could disrupt marine ecosystems, degrade ecosystem services, and affect human well-being. Hence, the present findings call for a reassessment of the environmental hazards posed by OC and BP, driving mitigation efforts and sustainable management to protect bivalve populations. Moreover, given the reported effects and the potential presence of BP in aquatic environments, regular monitoring of its concentrations is essential.

## 5. Conclusions

Octocrylene (OC) and benzophenone (BP) affect the early life stages of Pacific oysters similarly, inducing abnormal development in D-shaped larvae at environmentally relevant concentrations. However, no changes in DNA integrity or swimming velocity were detected. Development was the most sensitive endpoint, with all OC and BP concentrations being harmful.

The multidimensional approach and highly sensitive endpoints used in this study underscore the importance of evaluating contaminants’ toxicity during early life stages to predict population-level impacts. These results emphasize the need to reconsider the inclusion of these compounds, or their maximum allowable levels, in cosmetics and personal care products given their ecotoxicological hazards.

## Data Availability

Data will be made available on request.
